# Evaluation of the Function of Probiotics, Emphasizing the Role of their Binding to the Intestinal Epithelium in the Stability and their Effects on the Immune System

**DOI:** 10.1186/s12575-021-00160-w

**Published:** 2021-12-01

**Authors:** Nahid Javanshir, Golsa Nayeb Ghanbar Hosseini, Mahdieh Sadeghi, Ramtin Esmaeili, Fateme Satarikia, Gholamreza Ahmadian, Najaf Allahyari

**Affiliations:** 1 Department of Industrial and Environmental Biotechnology, National Institute of Genetic Engineering and Biotechnology. (NIGEB), P.O. Box: 14155-6343, Tehran, Iran; 2 Department of Microbial Biotechnology, Tehran University, Tehran, Iran; 3 Department of Science, Islamic Azad University - Parand Branch, Parand, Iran; 4 Payame Noor University, East Tehran Center, Tehran, Iran; 5 Department of Biological Sciences, Tarbiat Modares University, Tehran, Iran

**Keywords:** Probiotics, Functional mechanisms, Binding, Sortase, Immune system regulation

## Abstract

Due to the importance of using cost-effective methods for therapeutic purposes, the function of probiotics as safe microorganisms and the study of their relevant functional mechanisms have recently been in the spotlight. Finding the mechanisms of attachment and stability and their beneficial effects on the immune system can be useful in identifying and increasing the therapeutic effects of probiotics. In this review, the functional mechanisms of probiotics were comprehensively investigated. Relevant articles were searched in scientific sources, documents, and databases, including PubMed, NCBI, Bactibace, OptiBac, and Bagel4. The most important functional mechanisms of probiotics and their effects on strengthening the epithelial barrier, competitive inhibition of pathogenic microorganisms, production of antimicrobials, binding and interaction with the host, and regulatory effects on the immune system were discussed.

In this regard, the attachment of probiotics to the epithelium is very important because the prerequisite for their proper functioning is to establish a proper connection to the epithelium. Therefore, more attention should be paid to the binding effect of probiotics, including sortase A, a significant factor involved in the expression of sortase-dependent proteins (SDP), on their surface as mediators of intestinal epithelial cell binding. In general, by investigating the functional mechanisms of probiotics, it was concluded that the mechanism by which probiotics regulate the immune system and adhesion capacity can directly and indirectly have preventive and therapeutic effects on a wide range of diseases. However, further study of these mechanisms requires extensive research on various aspects.

## Introduction

Probiotics have a variety of applications in different fields. Today, probiotics are used as a treatment and prevention method in many diseases and disorders. Their preparation and application for the health of the host are evolving. The food industry has also focused on using probiotics in fermented dairy products following a time of safe use and gaining more knowledge about their positive impact on human health [1]. Tolerance of gastrointestinal conditions, mucosal adhesions, and deprivation of competition are the basic aspects of probiotic selection [2] The mechanisms by which probiotics work are unknown. The effect of probiotics on pathogenic microorganisms is related to several mechanisms, including antimicrobial secretion, competitive adhesion to epithelium and mucosa, reinforcement of intestinal epithelial barrier, and immune system regulatory impact [3].

**Fig. 1:**
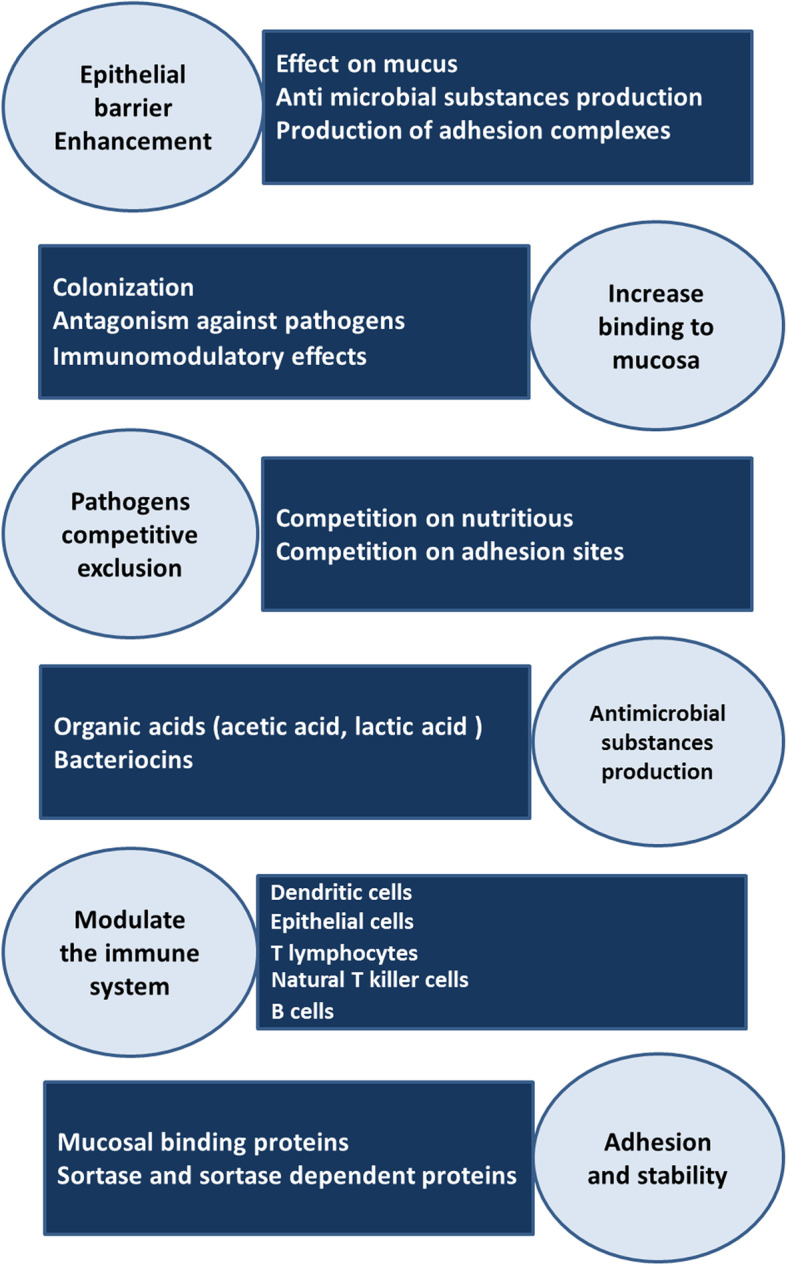
Major mechanisms of action for probiotics

Figure 1 shows major mechanisms of action for probiotics. Results that are supported by evidence from human experiments and animal models show the therapeutic potential of probiotics against several diseases [4]. It should be noted that the probiotic effects of different strains are not the same. Therefore, the health benefits attributed to one strain, also in one species, may not necessarily apply to another strain [5].

## Enhanced Epithelial Barrier

The epithelium of the intestine is constantly changing in continuous interaction with the dynamic luminal. The protection of an organism against pathogens is dependent on the defense mechanisms as well as intestinal barrier function. The intestinal barrier defense includes the function of the mucosal layer, production of antimicrobial peptides, secretion of IgA, adhesion mechanism, binding of beneficial bacteria residing in the intestine to the epithelial layer, and the formation of an epithelial binding complex [6]. When the epithelial barrier is weakened, inflammatory reactions that may lead to intestinal disorders can occur due to the penetration of intestinal mucosa by bacterial antigens and food allergens [7]. The utilization of probiotics can restore intestinal barrier function. The mechanisms of probiotics that enhance intestinal barrier function have not been thoroughly investigated, but it has been suggested that increased expression of genes contributing to cell signaling [8] is one of the potential mechanisms for increasing intestinal integrity.

The regulatory effect of *Lactobacillus* strains on several genes which encode binding proteins, such as Cadherin and βcatenin, has been observed in the T84 cell barrier model. Meanwhile, the phosphorylation of binding proteins and the frequency of the expression of protein kinase C delta (PKCδ) isoforms are greatly affected by the incubation of *Lactobacillus* strains with intestinal cells [9].

One of the roles of probiotics is repairing the epithelial barriers following an injury. *Escherichia coli* Nissle EcN1917 prevents the mucosal barrier dysfunction caused by Enteropathogenic *Escherichia coli* and repairs T84 and Caco-2 mucosal cells. This influence is promoted by the binding proteins ZO-2 and PKC protein kinase expression, which regenerate the strong binding complex [10]. Two strains of *Lactobacillus casei* DN-114001 and VSL3 can maintain intestinal barrier function through similar mechanisms [11]. In addition, the *Lactobacillus casei* VSL3 strain protects the epithelial barrier, which is the most prominent feature of this *Lactobacillus* strain.

Moreover, the *Lactobacillus* strain has been reported to play a role in activating P38 signaling pathway and regulating extracellular kinases by increasing the expression of proteins with strong binding [12]. It should be noted that the relationship between different levels of proinflammatory cytokines and intestinal penetrability depends on factors such as intestinal problems and disorders. Another function of probiotics is to protect and prevent epithelial damage caused by cytokines [13]. Apoptosis of intestinal epithelial cells as a consequence of the disruption of epithelial integrity and increased levels of inflammatory cytokines are two main pathological factors in inflammatory bowel diseases. P40 and p75, two soluble proteins produced by *Lactobacillus rhamnosus* GG, promote IEC homeostasis. By controlling the signaling pathways, P40 and p75 prevent cytokine-induced cell apoptosis in the intestinal epithelium of mice and humans [14]. They also modulate the expression of a proliferation-inducing ligand (APRIL) in the epithelium by transactivating the epidermal growth factor receptor (EGFR). In the mouse IECs, this pathway decreases cytokine-induced apoptosis and promotes IgA development. Another mechanism by which these two proteins stimulate the proliferation and viability of intestinal epithelial cells is the stimulatory effects on the IEC for the production of heat shock proteins Hsp72 and Hsp25, which in turn play a vital role in protecting strong binding proteins. Using a phosphatidylinositol-3-kinase-dependent (PIK3) mechanism, it also activates the Akt pathway. Meanwhile, P40 and P75 can prevent colitis and promote intestinal development. In addition, P40 and P75 prevent the disruption of tight junctions by H2O2 through the protein kinase C (PKC)-dependent mechanism [15]. Table 1 shows some probiotics and their mechanism of action to enhance the epithelial barrier.

**Table 1:** Probiotic strains and their mechanism of action to enhance epithelial barrier

Strain	Mechanism	Ref.
*Lactobacillus plantarum* ZLP001	Encoding genes related to antioxidative capacity (ClpP, HslV, trxA, trxB, tpx, nox2, npr, aspB).	[16]
	Maintaining epithelial integrity and preventing *Enterotoxigenic Escherichia coli* (ETEC)-induced gut permeability.	[6]
*Bacillus amyloliquefaciens* SC06	Increasing the intestinal epithelial cell barrier and immune function by improving intestinal mucosa structure, tight junctions, and activating the TLRs signaling pathway.	[17]
*Escherichia coli Nissle* 1917	Regulating the expression of tight junction proteins in the intestinal epithelial cells (IECs) (upregulation and redistribution of the tight junction proteins ZO-1, ZO-2, and claudin-14).	[18]
*Lactobacillus acidophilus Bifidobacteria bifidum Bifidobacteria infantum*	Modulating the gut microbiota and reducing colon cancer. Decreasing tumor incidence, multiplicity/count, and volume via enhanced TLR2-improved gut mucosa epithelial barrier integrity and suppression of apoptosis and inflammation.	[19]
*Clostridium butyricum*	Attenuating bacteria-induced intestinal damage and increasing the expression level of muc-2 and ZO-1 in the intestine and intestinal epithelial cells.	[20]
*Bifidobacterium infantis* *Lactobacillus acidophilus*	Protecting the intestinal barrier against IL-1β stimulation by normalizing the protein expression of occludin and claudin-1 and preventing IL-1β–induced NF-κB activation in Caco-2 cells, which may be partly responsible for the preservation of intestinal permeability.	[21]
*Bacillus subtilis* CW14	Treatment of *Bacillus subtilis* CW14 mitigates the tight junction injury by improving ZO-1 protein expression and reduced apoptosis s induced by ochratoxin A (OTA)	[22]
	Protects the ZO-1 protein by activating the TLR signaling pathway and reduces OTA damage by down-regulating the death receptor genes and up-regulating the DNA repair genes.	
*Bifidobacterium bifidum*	Strengthening of the intestinal epithelial tight junction prevents epithelial barrier disruption induced by TNF-α.	[23]
*Lactobacillus rhamnosus* GG	Protects the intestinal mucosa of rats from pepsin-trypsin-digested gliadin (PTG)-induced damage by preventing the reduction of the expression of the intercellular junction proteins.	[24]

## Increased Attachment to the Mucosa of the Intestine

Colonization is one of the main factors in the bacterial host interactions which occur following the bacterial adhesion to the host mucosa as a precondition [25].

Adhesion of probiotics to the intestinal mucosa is also necessary to regulate the immune system and antagonism against pathogens [26]. Consequently, adhesion is the main prerequisite for the selection of probiotic strains and is attributed to probiotic mechanisms. The surface determinants that communicate with IECs and mucosa are also present in LAB. A significant part of the mucosa consists of glycoprotein compound complexes secreted by the IECs, which have a vital role in preventing pathogenic bacteria attachment to the epithelium [27].

This interaction suggests that there may be a correlation between probiotic bacteria surface proteins and the pathogen’s competitive exclusion in the mucosa [28].

As discussed, several surface proteins of *Lactobacillus* promote the mucosal adhesions and form a surface binding in bacterium with the mucosal layers of the host cell, in which proteins, polysaccharide compounds, and lipoteichoic fatty acids play an important role [29] .

Mucus-binding proteins (Mub) are the most intensively researched surface proteins produced by *Lactobacillus reuteri* [30]. In the *Lactobacillus* strains, surface-dependent secretory proteins play a key role in mucosal adhesion [31]. It has also been reported that *Bifidobacterium animalis* subsp. lactis and *Bifidobacterium bifidum* have surface proteins that have a role in interaction with human plasminogen or enterocytes.

Surface proteins have a role in bacterial colonization in the human intestine by degrading the extracellular matrix of cells or by facilitating close contact with the epithelium [32]. Increased production of antimicrobial proteins such as alpha- and beta-defensins, catalysidins, type C lectins, and ribonucleases is the main host response to pathogenic bacterial invasion and serves as the host’s first line of chemical defense [33]. Enzymatic attacks on the cell wall structure or disruption of bacterial membranes by the secreted peptides and antimicrobial proteins are another line of defense by host cells that kill pathogenic bacteria. Several enzymes which act as an anti-microbial peptide (AMPs) expressed by paneth cells attack bacterial membranes. Lysozyme hydrolyzes the glycosidic bond of cell wall peptidoglycans [34], and phospholipids of the bacterial membrane are hydrolyzed by the phospholipase A2 [35]. In lactic acid bacteria, microbial adhesion often involves passive forces such as electrostatic interactions, hydrophobic interactions, steric forces, lipoteichoic acid, and some structures such as external lectin-based phenomena.

Although different types of proteins associated with the pathogenic bacterial binding have been identified, all the factors that affect the adhesion of probiotics have not been investigated yet.

The functional importance of different components of the mucosal layer and the diverse interaction of the mucosal layer, epithelial cells, and microbiota such as probiotics with the innate and acquired immune systems must be identified and analyzed in further studies [36].

## Competitive Inhibition of Pathogenic Microorganisms

The term “competitive exclusion” was first expressed in an experiment in which different species of bacteria competed to bind to receptors in the intestinal tract, but one species of bacteria was more able to bind to the receptors than other species and thus prevented the binding of other species. Bacteria use mechanisms such as creating a competitive microecology, destroying other bacterial receptor sites, producing and secreting antimicrobials and selective metabolites, and reducing nutrients competitively to eliminate the growth of other species. Bacterial adhesion potential to mucin by surface proteins contributes to the antagonistic function of probiotic species against the adhesion of gastrointestinal pathogens [37]. Evidence shows that a broad spectrum of pathogens such as *Salmonella, Escherichia coli*, *rotavirus, Listeria monocytogenes,* and *Helicobacter pylori* are inhibited by *bifidobacteria*. Production of antimicrobial substances and stimulation of ICEs are two probiotic mechanisms to inhibit the adhesion of pathogens [38].

Competitive inhibition of intestinal bacteria focuses on the interactions between bacteria and host, the purpose of which is to fight for accessible nutrients and epithelial attachment sites. Bacteria can also adjust their ecosystem to achieve strategic superiority, making it unsuitable for the competitors’ lives. The development of antimicrobial metabolites like organic acids (lactic acid and acetic acid) is an instance of environmental change [39]. Data from in vitro experiments on human or animal mucosa have shown the impact of lactic acid bacteria (LAB) on the competitive exclusion of pathogens. *Lactabacillus rhamnosus GG* has special adhesion properties that prevent the colonization of Enterohemorrhagic *Escherichia coli* (EHEC) in the human intestinal cell line. The mechanism of binding to surface glycoproteins and glycolipids of IECs is used in pathogens such as EHEC using mannose-sensitive type 1 fimbriae. The probiotic strains of *Lactobacillus* and *Bifidobacterium* prevent pathogen binding to the IEC by binding to the same receptor sites [40].

## Production of Beneficial Metabolites

A health-promoting mechanism in probiotics is the production of compounds with a molecular weight below 1000 Da, such as organic acids, as well as antibacterial agents called bacteriocins, which have a molecular weight of more than 1000 Da [3]. Acetic and lactic acids as organic acids have a significant restraining effect on gram-negative bacteria and act against pathogenic bacteria as one of the antimicrobial compounds of probiotics [41].

Inside the bacterial cytoplasm, the undecomposed form of organic acid binds to the bacterial cell and decomposes, resulting in a decrease in intracellular pH or intracellular build-up of ionized forms of organic acids that can induce the death of pathogens [42].

Small AMPs and bacteriocins are two of LAB’s important antimicrobial peptides. Bacteriocins developed by gram-positive bacteria such as LAB include lactacin B produced by *Lactobacillus acidophilus*, plantaricin produced by *Lactobacillus plantarum*, and nisin produced by *Lactobacillus lactis*. However, a group of bacteriocins acts against oral pathogens. Common mechanisms in bacteriocin function include target cell destruction by cavity development or cell wall synthesis inhibition in pathogens. It has also been demonstrated that *Bifidobacterium* has lethal functions against many pathogenic bacteria, including *Escherichia coli* C18455 and *Salmonella enterica typhimurium* SL1344. Production of a low molecular weight lipophilic molecule is the cause of this action. In addition, an important compound identified to be effective against gram-negative bacteria is a low molecular weight protein called BIF, expressed by *Bifidobacterium longum* BL198. This protein hinders the *E. coli* binding to the epithelial cell line in human [43].

Some probiotics produce metabolites that have an inhibitory effect on fungi and other bacteria. *Lactobacillus* can produce various antifungal agents such as mevalonolactone, benzoic acid, Short-chain fatty acids (SCFAs), and methylhydantoin. *Lactobacillus coryniformis* is also able to produce protein compounds with antifungal properties [44].

SCFAs such as acetic, propionic, and butyric acid are important bacterial metabolites of the intestine with crucial functions in the host’s health. Several factors impact the concentration of SCFAs in the intestine, such as the population of intestinal bacteria, environmental effects, genetic factors, and diet. Based on the studies, there is an interaction between SCFAs and diseases such as inflammatory bowel disease (IBD), type 2 diabetes (T2D), obesity, autoimmune disorders, and bacterial infections. Table 2 presents SCFAs produced by probiotics and their beneficial effects.

**Table 2:** SCFAs produced by probiotics and their beneficial effects

Short-chain fatty acids	Effect of SCFA	Producing Bacteria	Ref.
Lactic acids	Maintaining intestinal and immune homeostasis As a mediator in the microbiota–gut–brain axis crosstalk regulating pH, Increasing the absorption of calcium, iron, magnesium, Anti-inflammatory activity (inhibiting NFκB macrophages) Inhibit the development of pathogenic microorganisms competing for colonization sites	Lactic acid bacteria (LAB) Lactobacilli, Bifidobacteria, Enterococci, Streptococci, Eubacterium	[39, 45–48]
Acetic acid	Key factor in the metabolism of carbohydrates and fats and synthesis of cholesterol Maintaining intestinal and immune homeostasis	LAB, Acetic acid bacteria (AAB), *Acetobacter* spp.	[49, 50]
Propionic acid	Inhibition of gluconeogenesis and cholesterol synthesis in the liver Maintaining intestinal and immune homeostasis Antibacterial and anti-inflammatory effects against pathogens	LAB Propionic acid bacteria (PAB) Propionibacterium	[51, 52]
Butyric acid	Anti-inflammatory effect Main source of energy for intestinal epithelial cells Immunoregulatory effect on intestinal epithelial cells and other mucosal cell populations Stimulating the expression of the MUC2 gene in cell lines. Production of mucin inhibiting tumor development and inducing the process of apoptosis	LAB, Bacteroidetes, Firmicutes	[47, 53–57]

## Probiotics and the Immune System

The first line of defense against pathogens is the innate immune system, although its ability to detect antigens is not specific. Several different types of cells make up the innate immune system, and these cells are the first cells to encounter and respond to pathogens and their metabolites [58]. Epithelial cells and dendritic cells (DCs) are the most common innate immune cells mentioned in studies on probiotics and their relationship with the immune system [38].

The main cells of the innate immune system are phagocytic cells such as macrophages, neutrophils, dendritic cells, monocytes, natural killer (NK) cells, and mast cells [39]. One study found that consuming yogurt fermented with *Lactobacillus*. *bulgaricus* OLL1073R-1 increased the activity of NK cells and reduced the risk of colds in the elderly [59]. Other studies have demonstrated that *L. bulgaricus* OLL1073R-1 and its secretory polysaccharides enhance the activity of the immune system, which in turn activates the NK cells. Therefore, prevention of respiratory infections caused by respiratory viruses or influenza is achieved by using *L. bulgaricus* OLL1073R-1 or its products [60, 61].

In another study of physically active individuals (university athletes), *Lactobacillus casei* Shirota decreased the plasma antibody titers against cytomegalovirus and *Epstein-Barr* virus (EBV) [62]. However, there was no substantial difference between the group receiving *Lactobacillus casei* shirota and the control groups in the occurrence of gastroenteritis by *Norovirus* in the long-stay elderly at a healthcare facility [63].

Although the possible mechanism of action of *Lactobacillus casei* shirota is still debated, a study has reported that the function of NK cells, one of the first defense mechanisms against viral infections, is modulated by *Lactobacillus casei shirota* [64]. The link between acquired and innate immune systems is created by DCs, macrophages, and monocytes, as they act professionally as antigen-presenting cells (APCs). Probiotics possess antiviral effects, while the NK cells have increased cytotoxic ability and phagocytosis of macrophages is growing. Through the secretion of tumor necrosis factor alpha (TNF alpha), components of the gram-positive bacterial cell wall, including lipoteichoic acid (LTA), can stimulate nitric oxide (NO) synthase as a mechanism of virus-infected cell death by macrophages and also enhance the configuration of essential phagocytosis receptors like FcγRIII (CD16) and toll-like receptors (TLRs) [65, 66]. These cells are important in that they function to initiate acquired immune responses because primary T cells provide a response to APC-presented antigens and cytokines secreted in acquired immunity and differentiate particular subtypes of CD4 + T cells (Th1, Th2, or Th17 cells) [67, 68]. Furthermore, for the acquired immune responses to be successful, the interactions between T cells and APC are critical [39].

The inhibitory impact of *Lactobacillus rhamnosus* GG on experimental rhinovirus infections has been assessed in healthy volunteers. Rhinovirus was inoculated intranasally to volunteers, followed by ingestion of *L. rhamnosus* GG for 6 months. The incidence and severity of cold symptoms and the number of individuals with rhinovirus infection were lower than those of the control group [69].

In another study, children with rotavirus-induced diarrhea who received LGG showed a significant increase in serum IgG immunoglobulin levels after the intervention. Meanwhile, with LGG administration, a significant improvement in intestinal permeability was observed in children with cryptosporidium-induced diarrhea [70]. *L. rhamnosus* GG has also been suggested to modulate innate and adaptive immune responses, specifically against gastrointestinal pathogens, leading to increased serum IgG and secretory IgA levels targeting intestinal pathogens, including rotavirus [47]. A plausible pathway for host microorganisms to interact with the surface of the intestinal mucosa and gut-associated lymphoid tissue (GALT) is a connection between IECs and bacteria and their metabolites. Moreover, IECs and APCs play a role in the innate immune system. The activity of IEC cells is regulated by commensal and probiotic bacteria, which enables IEC to affect the immune cells such as DCs, macrophages, and intra-peritoneal lymphocytes [71, 72]. NF - κβ activity in IEC cells has been reported to be inhibited by intestinal commensal bacteria in cases of *Salmonella typhimurium* and *Salmonella pullorum*. The mechanism of this inhibition is to inhibit the nuclear transfer of NF - κβ protein by inhibiting Ikβ-α ubiquitination [73, 74]. This mechanism has also been shown to decrease the expression of inflammatory cytokines and their mediators, including IL8 [75]. IECs also can restrain the high load of common bacteria in the distal region of the human intestine [39].

### Dendritic Cells (DCs) and Probiotics

DCs are primary cells that function as microbial ligand sensors by activating innate immune receptors (e.g. TLRs and C-type lectin receptors (CLRs)). The signaling pathways are activated by bacterially derived molecules permitting changes in the phenotypes of DCs and secretion of cytokine, which can be underpinned by the integration of immune functions with microbial and host metabolism. An important immune-regulating activity performed in the intestinal DC is the metabolism of vitamin A to retinoic acid. Recent research has shown that some probiotics in the small intestine can induce this metabolism in human and mouse DCs [76, 77].

In addition, it has been reported that in addition to vitamin A metabolism, the induction of another DC metabolic enzyme, heme oxygenase-1 (HO-1), is essential for the induction of mucosal T regulatory (Treg) cells within the mesenteric lymph nodes by *Lactobacillus rhamnosus* [78]. In addition to metabolites, bacterial cell wall components are also involved in the regulation of the immune system by DCs. The presentation of segmented filamentous bacterial antigens by intestinal CD11cþ DCs, which is dependent on histocompatibility complex (MHC)-II, is essential for the induction of TH17 lymphocytes [79]. Moreover, a direct association between specific fragments of *Bacteroides fragilis*, capsular polysaccharide A (PSA), and mouse plasmacytoid DCs through TLR2 has been demonstrated. Plasmacytoid dendritic cells have been shown to express proteins that protect the gut against colitis as well as IL-10 secretion by CD4þ cells after exposure to PSA [80].

An exopolysaccharide from *Bacillus subtilis* is associated with TLR4 and MyD88 signaling and protects the gut from inflammation induced by *Citrobacter rodentium* [81]. Dietary fiber fermentation in the colon, including prebiotics, results in the production of SCFAs [82]. The Pathogenesis of inflammatory colorectal cancer, obesity, bowel disease, allergies, and type II diabetes may lead to abnormal production of these metabolites owing to dysbiosis and dietary issues [83]. Butyrate, acetate, and propionate are likely to be more effective in stimulating the immunomodulatory effects of all SCFAs [84]. Based on a recent study, butyrate, which helps induce IL10-secreting T cells and Treg cells, induces the regulatory activity of the DCs, which is mediated on colonic DCs and macrophages by the G-protein-coupled receptor, Gpr109a [85, 86]. It has been shown that Butyrate induces IL23 secretion by mouse DCs, which activates the TH17 response under certain conditions and also increases mucosal histamine levels in patients with inflammatory bowel disorder and intestinal syndrome [87]. It can reduce the secretion of proinflammatory chemokines and cytokines that are stimulated by TLR-stimulated DCs, albeit raising IL10 production [88].

Activation of histamine 2 receptor (H2R) on DCs was initiated by histamine exerting its effect, and the signaling mechanism needed direct protein exchange by cAMP (Epac) and cyclic adenosine monophosphate (cAMP). Experiments on H2R-deficient mice have shown quick weight loss and raised Peyer’s patch cytokine secretion, being more intense by a histamine-secreting *Lactobacillus strain* [89]. Microorganisms have different effects on the phenotype and function of DC. Certain bacteria stimulate the immune system, while others stimulate tolerant reactions. What determines the impact of a particular bacterial strain on DC function has remained unknown so far. However, specific patterns or mosaics of microbial-related ligands can affect the outcome of certain compositions of receptor-mediated cell-signaling pathways. Using probiotic-based therapy to regulate DC maturation can modulate the immune response. Kwon et al. [90] reported that regulatory DC drives the generation of CD41 Foxp3+ Treg cells following the administration of combined probiotic strains in mice by expressing high levels of IL10, transforming growth factor, indoleamine 2, 3-dioxygenase (IDO), and cyclooxygenase-2 (COX-2).

Tryptophan metabolites have an important role in the microbiota- intestinal -brain axis regulation in physiological and pathological conditions. The intestinal microbiota has an impact on controlling tryptophan metabolites. Therefore, modulating tryptophan metabolism by positively altering the microbiota with prebiotics or probiotics may be an effective treatment. N′-Formylkynurenine is a mediator in tryptophan catabolism whose synthesis is catalyzed by IDO [91].

By depleting autoreactive T cells and inducing Treg cell responses, IDO-expressing DC contributes to the generation and maintenance of peripheral tolerance [92]. IDO activity is stimulated by the ability of certain microbes in addition to the expression of IL10 by DC, which may be necessary to develop tolerance and to generate a regulatory immune response. Perhaps significantly maintaining a non-clinically responsive condition following allergen exposure in atopic individuals is linked with elevated IL10 production and IDO activity [93]. However, after exposure to bacteria, several components of the immunoregulatory system contribute to the tolerogenic potential in DC. Recently, it has been shown that feeding mice with *L. rhamnosus* JB-1 leads to an improvement in DC activity and regulatory/tolerogenic phenotype in the mesenteric lymph nodes (MLN) [78].

### Epithelial Cells and Probiotics

Epithelial cells are indisputably involved in nutrient uptake. One of the defensive barrier mechanisms is the synthesis and secretion of antimicrobial peptides like defensin and cathelicidin. Probiotic strains are involved in the differential regulation of defensin expression and protein secretion, which itself is affected by local inflammatory mediators [94–96].

Autophagy can be seen as an essential adaptive response to stress to promote cell viability, which is necessary to maintain the epithelial barrier. In this regard, some *Bifidobacteria* have been recently identified in an intestinal cell line that facilitates autophagy [97].

It has now been shown that autophagy is essential for proper intestinal function, and its impairment is associated with loss of intestinal homeostasis. Gram-negative bacterial lipopolysaccharide is one of the leading causes of pathogenic autophagy leading to IEC cytotoxicity. Chaobiun Han et al. [98] reported that probiotic-mediated autophagy could prevent this cytotoxicity and be used as a new mechanism to protect epithelial cells. It has been indicated that probiotics increase intestinal mucin production by goblet cells, and mucin covers the gastrointestinal tract and acts as a major protective barrier. In a recent study, p40 *Lactobacillus* GG was shown to transactivate epidermal growth factor receptors to stimulate mucin production [99]. In addition, local inflammatory reactions which can destroy the intestinal epithelial barrier are caused by strong responses of the epithelial cells to microbial ligands [100–102]. The effect of the chemokine response is not the same in every probiotic strain, and on the other hand, the expression of certain chemokines can be increased or decreased by a single probiotic strain. To exemplify, *Bifidobacterium bifidum* PRL2010 increases CCL19 cytokine expression but inhibits CCL22 expression, which supports the idea that probiotics induce strain-specific chemokine-specific responses [103]. Moreover, secretion of proinflammatory mediators, production of antimicrobial peptides, and epithelial barrier function can be modulated by their prebiotics or SCFAs [104, 105]. The adaptive immune system receives different signals from the innate immune cells to extend an adequately regulated lymphocyte response to bacterial and metabolic factors. Recent studies have indicated the role of probiotics and prebiotics on NKT cells, Tregs, effector T cells, and B cells [38]. A balance between immune tolerance and inflammation can regulate the crosstalk between the intestinal microbiota and innate and adaptive immune cells [106].

### T Lymphocytes and Probiotics

It has been shown that there is an association between increased Tregs function and the positive impact of probiotics, symbiotics, and prebiotics on illnesses like allergies or colitis [67, 107]. Based on the results of various studies, probiotics may regulate the secretion of vital immune modulators, especially by innate immune cells like DCs, thereby causing specific regulation of T lymphocytes [108, 109]. Different strains of lactobacilli can have different regulatory effects on the immune system response. Different pathogen-associated molecular pattern molecules (PAMPs) expressed by the lactobacilli strains can induce cytokine release regulation and will be recognized by the related pattern recognition receptors (PRR) on APCs. The bacterial-dependent cytokine setting obtained will indicate an important signal for T cells, which ultimately determines the regulation of the following T cell response.

Table 3 shows *lactobacillus* strains and their capability of inducing Th1, Th2, Th17, or T regulatory cell responses [110].

**Table 3:** *Lactobacillus* strains and their ability to regulate T cells

Strain	Regulate	T cells	Ref.
*L.casei* DN-114001	IL12	Th1	[111]
*L.paracasei* Z11			[112]
*L.acidophilus* X37			[113]
*L.salivarius* A6			[114]
*L.gasseri* 19,992			[115]
*L.johnsonii* 33,200			[92]
*L.reuteri* (ATCC 23272)			[116]
*L.reuteri* DSM 12246	IL12	Th2	[117]
*L.reuteri* 5289			[78]
*L.plantarum* DN-121			[118]
*L.rhamnosus* GG	IL23	Th17	[119]
*L.rhamnosus* Lcr35			[120]
*L.casei* NIZO B255	IL10	Treg	[121]
*L.reuteri* ASM20016			[122]

As a result, owing to their regulatory ability, probiotics are facinating candidates for the treatment of many inflammatory diseases, including irritable bowel syndrome (IBS), allergies, and other immune-mediated diseases. Since particular probiotic bacteria may be associated with various immunomodulatory properties, it may be possible to use the strain to enhance the therapy of Th-mediated intestinal pathologies or modulate the immune response if more detailed information is available on the immunomodulatory effects of certain bacteria. Moreover, some probiotic bacteria, or their molecular components may be beneficial as vaccine adjuvants by inducing DCs to enhance type I T cell response, due to the reaction of DC-produced inflammatory cytokines to bacteria [123, 124]. Certain probiotics have been reported to increase the number or function of Tregs. Consumption of *Bacillus infantis* 35,624 brings about two distinct outcomes. In psoriasis patients with chronic fatigue syndrome, or patients with ulcerative colitis, this leads to a decrease in the level of serum proinflammatory biomarkers such as C-reactive protein, which may be mediated by the number of Tregs. On the other hand, in healthy volunteers, it can increase the level of Foxp3þ, which is involved in regulating the immune response in peripheral blood lymphocytes [125].

### Natural Killer T (NKT) Cells and Probiotics

Although CD1dþ epithelial cells act against intestinal inflammation, NKT cells are the primary mediators of intestinal inflammation and bone-marrow-derived CD1dþ cells mediate the induction of pathogenic NKT cells [126]. It NKT cells have been suggested to be directly affected by the intestinal microbiome and activated by probiotic antigens [127, 128].

Childs et al. reported that the use of symbiotic supplements, including xylo-oligosaccharide in combination with *Bifidobacterium animalis*, reduced the expression of CD16/56 cell surface markers in NKT cells and IL-10 secretion in a healthy volunteer, while this symbiotic reduced CD19 expression in B cells. Besides, it can also decrease IL10 secretion in response to lipopolysaccharide in peripheral blood mononuclear cells [128]. The practical outcome of altered activation in NKT by the human microbiome has not yet been determined.

### B Cells and Probiotics

The importance of B lymphocytes in humoral immune responses is evident through their secretion of antigen-specific antibodies. B cells limit aggressive immune reactivity and regulate immune responses principally via IL10 in the experimental models of allergic inflammation, tolerance, and infection [129]. Microbiome modulation based on B cell was demonstrated in mice deficient in IgA. Lundell et al. also found another mechanism related to IgA concerning intestinal microbiota maturation in mice [130]. Another research group has proposed the activation of naive B cells in the gut, which overlaps the human intestinal microbiota production, and the presence of a significant number of IgGþ and IgAþ B cells in the gut, which confirms the role of B cells as immunomodulators [131]. Oral administration of *Lactobacillus gasseri* SBT2055 has been found to trigger IgA production and boost the number of IgAþ cells in the lamina propria and Peyer’s patches.

Simultaneous stimulation of B cells with B-cell activating factor and *L. gasseri* SBT2055 was found to increase the induction of IgA production.

The main group of antibodies found in the body’s secretory fluids such as saliva, mucus, and tears is salivary immunoglobulin A (SIgA). SIgA has a role in mucosal surface, including host defense against pathogens transmitted by the mucosa, which control the quantity and quality of commensal microbiota composition by the host [132]. In a human study, supplementation of *B. animalis* with xylooligosaccharide reduced the expression of CD19 in B cells, suggesting that immune system modulations are caused by correctly chosen probiotics, prebiotics, or a combination thereof. The results of research on the molecular mechanisms involved in immune cells show the important effect of SCFA. However, SCFAs are possibly one of many bacterial products that affect the immune system [133].

If a potential combination of probiotics and prebiotics is needed for better prevention and treatment of immune disorders, it is recommended to characterize probiotics and metabolites which have an impact on the immune system [134].

## Adhesion Mechanisms of Probiotics to Intestinal Mucosa and Stability

One of the significant features in selecting the type of probiotic bacteria is their ability to attach to the surface of the host gastrointestinal tract (GIT) [102]. The ability of probiotics to adhere to the surface of the gut can subsequently lead to the colonization of these bacteria in the gut, modulate and improve the function of the immune system, and also reduce autoimmune problems. These bacteria also contribute to the intestinal defense barrier against harmful bacteria or pathogens by eliminating or reducing them and improving metabolic functions [135, 136]. Probiotics act as a barrier by preventing the binding of pathogens and toxins to epithelial receptors. Further, in vitro experiments on intestinal cell lines have been commonly used to assess the antagonistic effects of probiotics on pathogens [137]. While the adherence ability of probiotics to the surface of the host intestine is not inherently a health advantage, the binding of probiotics to the intestinal mucosa by competing with binding to the host cell attachment sites may play a protective role in enteropathogens [138]. In addition, the adherence potential of probiotic bacteria to the GIT increases the possibility of contact with the host intestine, transient colonization, and their retention time in the gut to exert their beneficial effects [139].

This transient colonization, for instance, stimulates the local function of probiotic metabolites such as SCFAs as well as the effects of bacterial surface molecules on the immune system. These molecules function as ligands for the host intestinal epithelial receptors, resulting in the induction of signaling pathways. Moreover, prebiotics such as oligosaccharides can increase the probiotics’ adhesion ability [140]. *Lactobacillus* and *Bifidobacterium* are the most important gram-positive lactic acid bacteria that have common surface molecules such as surface layer proteins, Mub, and lipoteichoic acid, which have a crucial role in interacting with mucosal constituents [141, 142]. Intestinal surface bacterial adhesion can be initially induced by nonspecific contacts such as hydrophobic interactions, accompanied by the second phase of adhesion by specific cell wall constituents [143]. Surface adhesive proteins, including MucBP and Mub [144], are mucus-binding proteins. In many bacteria, including pathogenic species such as *Listeria monocytogenes* [145], a wide range of these proteins can be identified. Mucus binding proteins have also been found in lactic acid bacteria separated from the human gastrointestinal tract. On the other hand, the pili or fimbriae are also involved in the binding [146]. Other surface proteins such as surface layer proteins and fibronectin-binding proteins can play a role in the adhesion of bacteria to the intestinal mucosa in addition to mucus-binding proteins [147]. Fibronectin, a plasma-soluble glycoprotein, is found in the solid and insoluble form on cell surfaces and in the extracellular matrix. Therefore, fibronectin-binding proteins in both gram-negative and gram-positive bacteria can contribute to their binding to the intestine. The presence of these proteins in certain pathogens has been attributed to their pathogenicity, due to their ability to invade host epithelial cells [146]. The existence of fibronectin-binding proteins can also be beneficial for probiotics due to their improved ability to adhere to host cells. Surface layer proteins are extracellular paracrystalline proteins that cover the surface of some pathogenic bacteria and are the cell wall structural components that increase their pathogenicity [148]. The distribution and type of the surface layer proteins differ from one strain to another, but they tend to be important for the intestinal cell-probiotic bacteria adhesion. In addition, surface layer proteins can interact with host intestinal receptors to induce immune responses and also act as a regulatory factor in the immune system in probiotic bacteria [149]. The cell wall in gram-positive bacteria is a vital cell component that affects the function and survival of the bacterium. The responsibilities of the cell wall include facilitating interaction with the environment, creating a barrier against osmotic pressure, and preserving the stability of the structure. The gram-positive bacterial cell wall contains a large set of molecular structures which promote such interactions [150]. Meanwhile, surface proteins are greatly involved in establishing bacterial connections with the environment. Sortases are a group of membrane-associated transpeptidase enzymes in gram-positive bacteria that are responsible for binding a large set of secretory proteins to the surface of these cells via the identification of a motif preserved in these proteins to bind to the cell wall of gram-positive bacteria [151]. Sortase-dependent proteins (SDPs) play a key role in strengthening and improving the binding of probiotic bacteria, especially *Lactobacillus* species, owing to the pivotal role of sortase in binding its substrate to the gram-positive bacterial cell wall and the significance of proper binding to epithelial cells in improving probiotic bacterial function [152]. The first detected sortase, the sortase class A, was isolated from *Staphylococcus aureus* in 1999 [153]. Other groups of sortase have been discovered in the last decade. The name sortase is derived from its role in sorting the proteins passing through the secretory pathway [154]. After identifying the sortase enzyme, they were widely studied and considered medicinal targets due to their role in binding cell wall pathogenic factors in pathogens [155]. These enzymes are also involved in the polymerization of pili units in bacteria, which is one of the key factors that bind bacteria to surfaces [156]. An important feature of bacterial cell lining is the display of various surface proteins on the peptidoglycan layer, where these proteins can play a role in intestinal adhesion and pathogenicity [157]. Often, these surface proteins are covalently bound to the peptidoglycan layer of bacterial cell walls by a family of transpeptidase enzymes [158]. Sortase has different classes, including A, B, C, D, E, and F. As a housekeeping protein, class A sortase has a role in the covalent binding of surface proteins with the LPXTG motif to the cell wall of bacteria in gram-positive bacteria [159]. Class B sortase bind iron-absorbing proteins to the cell wall and is involved in iron homeostasis [160]. Class C sortase is involved in pili formation through polymerization of pili-forming units [161]. Class D sortase involves sporulation, and the function of F and E sortases, which are generally detected in actinobacteria, remains unknown.

### The Function of Sortase and SDPs in Probiotics

The gram-positive bacterial cell wall is a vital part of the cell that affects bacterial function and viability. The cell wall keeps the structure stable, creates a barrier against osmotic pressure, and facilitates environmental interaction. A variety of macromolecular structures that promote these interactions are present in the gram-positive bacterial cell wall. Teichoic acid, lipoteichoic acid, exopolysaccharide, enzymes, S-layer proteins, and other cell surface proteins are found in adhesions and pili-like structures, and are specifically associated with host binding [162]. Pathogenic bacterial surface proteins like protein A in *Staphylococcus aureus* and internalin A in *Listeria monocytogenes* have an important role in their infection and pathogenicity [155, 163]. Instead, the surface structures in probiotic bacteria have a key role in benefiting the host, which is placed on the surface of the bacteria during the surface display process. The surface display is a combination of two processes: protein targeting and protein binding to the extracellular membrane. Most proteins targeted for extracellular space travel through one of two pathways: the twin-arginine (Tat) pathway and the Sec pathway. The Sec pathway identifies unfolded proteins, which have a signal peptide in their N-terminal region, which contains a basic N domain, a hydrophobic H region, and a C domain that has a conserved site for signal peptidase cleavage. The secreted proteins are either transported out of the cell or bonded to the cell wall depending on the amino acid sequence in their C-terminal region. Proteins that bind to the membrane from the C-terminal region and are transported by the secretory pathway are in the *lactobacilli* group as a large part of membrane-bound proteins [164]. The Tat pathway, unlike the Sec pathway, transports folded proteins out of the cell [165]. This pathway, however, seems to be very unusual in lactic acid bacteria species. Covalent bonds or non-covalent interactions further bind the targeted proteins to the cell surface.

**Fig. 2:**
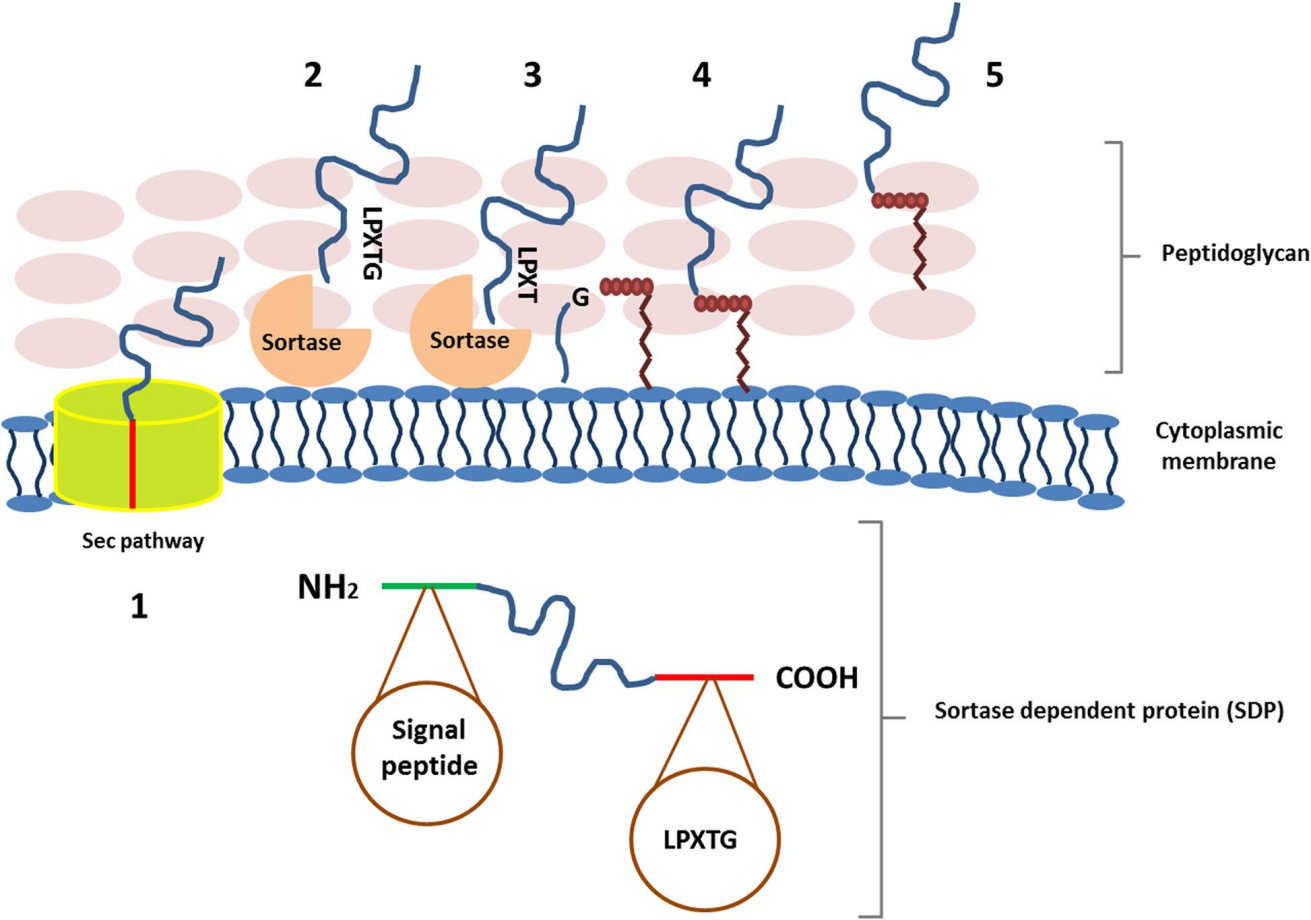
Sortase mechanism of action in binding SDPs to cell wall. **A** SDPs are distinguishable with The presence of an N-terminal signal peptide and a C-terminal LPXTG sorting signal, followed by hydrophobic and positively modified residues that facilitate membrane anchoring, distinguishes sortase substrates. **B** In a sequence of five processes, SDPs are linked to the cell wall;1. sec machinery recognizes the signal peptide on the SDPs and exports it to the cell’s exterior. 2: When sortase and the SDPs are in close proximity, sortase cleaves the SDP between the glycine and threonine residues with transpeptidase action.3 The sortase/SDP complex is dissociated by lipid II’s nucleophilic attachment and 4: Through contact with the pentapeptide cross bridge, it produces a lipid II intermediate.5: As part of regular cell wall construction, the sortase substrate is integrated into the cell wall

LPXTG-containing proteins or sortase-dependent proteins (SDPs) are a series of surface proteins that covalently bind to cell wall peptidoglycans after passing the membrane through the Sec pathway. In their C-terminal region, these proteins have a sorting signal with amino acid sequences of leucine (L), proline (P), threonine (T), amino acid (X), and glycine (G), which attach to the cell wall through an immobilization mechanism mediated by sortase A. Cell wall-SDPs attachment is achieved successfully not only by the role of the LPXTG motif but also by the presence of a signal peptide in the N-terminal domain and a positively charged tail and a hydrophobic region in the C-terminal domain. SDP is secreted by the signal peptide through the Sec pathway. The charged tail in the C-terminal region is required to bind the proteins covalently to the cell wall at the moment of leaving the cell. This temporary binding to the membrane by the C-terminal puts the sortase and its substrates in close proximity and embeds them in the membranes, allowing the sortase to perform its transpeptidase activity. The transpeptidase mechanism of sortase is then performed, which is essential for binding. The initial phase of the transpeptidase mechanism is the sortase substrate cleavage in the cleavage motif between threonine and glycine and the formation of the sortase-SDP complex. The nucleophilic acid attacks the resulting acyl- thioester bond formed between the two proteins, which eventually binds to the cell wall surface [150] (Fig. 2).

Members of the lactic acid bacterial group are known to be safe bacteria with health benefits. They have functions like maintenance of epithelial barrier function, reduction of symptoms of irritable bowel syndrome, and competitive inhibition of pathogens [166]. In many cases, the mechanism of these effects is unclear, and various factors are influential, but the presence of sortase and SDPs in LAB strains is considered an important factor in the development of the molecular mechanism of host-bacterial interactions. In addition, the mechanism of sortase in presenting the surface proteins to the cell wall is an interesting hypothesis in oral vaccine production using safe strains [167]. Genomic studies have shown that the presence of cell surface structures is responsible for the host-bacterial interactions. The role of different types of sortase in bacterial-host interaction makes it an attractive target for screening the genomes of various probiotic bacteria.

Studies have shown that a combination of two factors, the ability of SDP to bind to the intestinal surface and the modulation of the immune system by probiotics, can affect gastrointestinal conditions in the presence of pathogens [136, 168]. Recently, the role of sortase and SDPs has received attention beyond the range of pathogenic bacteria, and this enzyme is considered very important in the stabilization of surface proteins and the surface arrangement of cells in probiotic and non-probiotic bacteria. Studies have also used this mechanism of sortase to display antigens on the surface of lactic acid bacteria and other probiotic bacteria to develop safe oral vaccines. The idea that sortase can play a key role in bacterial physiology, including bacterial-host interactions, requires its investigation in various lactic acid bacterial species using new high- throughput genomic analysis tools [169, 170]. It has been reported that SDPs play an important role in the proper binding of probiotics, especially *Lactobacillus*, to intestinal epithelial cells and improve their probiotic function due to the role of sortase in their attachment to the probiotic bacterial cell wall. Based on current studies, it can be hypothesized that sortase and its substrates have a key role in bacterial physiology. This has led other researchers to identify and isolate new strains of *Lactobacillus* in their studies with different copies of sortase and its substrates, intending to find new strains with stronger probiotic properties.

## Conclusion

Probiotics have a significant capacity for the prevention of infections or the treatment of various diseases. However, some health findings on the effectiveness of probiotics are not yet conclusive and require more scientific data. The mechanisms of action of probiotics on the host health discussed in this review include modification of intestinal microbiota, effective binding for competitive inhibition of pathogenic microorganisms, boosting the intestinal epithelial barrier, and controlling the host immune system. However, further studies are needed to find other mechanisms of probiotic function. One of the mechanisms considered to be effective in bacterial-host interactions is the sortase and SDPs. In general, two factors, the ability of SDPs to bind to receptors on the intestinal cell surface and the immune system stimulation by probiotics, can affect gastrointestinal conditions when exposed to pathogens. Therefore, it is possible to predict the role of sortase and SDPs in the LAB to provide a proper binding to epithelial cells, to improve the functions of probiotic bacteria, to provide oral vaccines and drug delivery, etc. The mechanism of action of SDPs binding covalently to the cell wall is a successful strategy in surface display technology used by pathogenic gram-positive bacteria to use their host. The probiotic and commensal bacteria also use this strategy to positively interact with the host.

Gastrointestinal probiotics have a key role in the host immune response, strengthening the immune system, and improving the host’s health. The immune system can be greatly affected by probiotics owing to the immunogenic effects of probiotic bacteria and their ability to communicate with epithelial cells, DCs, lymphocytes, macrophages, and monocytes. Therefore, it may be useful to study the effects of probiotics on the immune system in combating and preventing many diseases. The right combination of probiotics and prebiotics has powerful effects on the immune system. Special attention has been paid to current data about molecular mechanisms underlying the impact of SCFA on immune cells. However, it might be useful to further study the bacterial strains and metabolites that affect immune function for the sake of the prevention and treatment of immune disorders and many diseases.

## Data Availability

Not applicable for this study.

## Abbreviations

AAB: Acetic acid bacteria
AMPs: Anti-microbial peptide
APCs: Antigen-presenting cells
APRIL: Proliferation-inducing ligand
cAMP: Cyclic adenosine monophosphate
CLRs: C-type lectin receptors
COX-2: Cyclooxygenase-2
DCs: Dendritic cells
EBV: Epstein-Barr virus
EGFR: Epidermal growth factor receptor
EHEC: Enterohemorrhagic *Escherichia coli*
ETEC: Enterotoxigenic *Escherichia coli*
G: Glycine
GALT: Gut-associated lymphoid tissue
GIT: Gastrointestinal tract
H2R: Histamine 2 receptor
HO-1: Heme oxygenase-1
IBD: Inflammatory bowel disease
IBS: Irritable bowel syndrome
IDO: Indoleamine 2, 3-dioxygenase
IECs: Intestinal epithelial cells
L: Leucine
LAB: Lactic acid bacteria
LTA: Lipoteichoic acid
MHC: Major histocompatibility complex
MLN: Mesenteric lymph nodes
Mub: Mucus-binding proteins
NK: Natural killer
NO: Nitric oxide
OTA: Ochratoxin A
P: Proline
PAB: Propionic acid bacteria
PAMPs: Molecular pattern molecules
PIK3: Phosphatidylinositol-3-kinase-dependent mechanism
PKC: Protein kinase C
PKCδ: Protein kinase C delta
PRR: Pattern Recognition Receptors
PSA: Polysaccharide A
PTG: Pepsin-trypsin-digested gliadin
SCFAs: Short-chain fatty acids
SDPs: Sortase-dependent proteins
SIgA: Salivary immunoglobulin A
T: Threonine
T2D: Type 2 diabetes
Tat: Twin-arginine
TLRs: Toll-like receptors
TNF alpha: Tumor necrosis factor alpha
Treg: T regulatory
X: Each amino acid
